# A novel reversible logic gate and its systematic approach to implement cost-efficient arithmetic logic circuits using QCA

**DOI:** 10.1016/j.dib.2017.10.011

**Published:** 2017-10-07

**Authors:** Peer Zahoor Ahmad, S.M.K. Quadri, Firdous Ahmad, Ali Newaz Bahar, Ghulam Mohammad Wani, Shafiq Maqbool Tantary

**Affiliations:** a Department of Computer Science, University of Kashmir, Srinager, Jammu and Kashmir, India; b Department of Computer Science, Jamia Millia Islamia, New Delhi, India; c Department of Electronics & IT, University of Kashmir, Srinager, Jammu and Kashmir, India; d Department of Information & Communication Technology, Mawlana Bhashani Science and Technology University, Tangail, Bangladesh; e Department of Physics, Sri Pratap College, Srinagar, Jammu and Kashmir, India; f Department of Physics, Govt Degree College Patan, Jammu and Kashmir, India

**Keywords:** QCA, F-Gate, Adder, Subtractor, Adder–subtractor, QCADesigner

## Abstract

Quantum-dot cellular automata, is an extremely small size and a powerless nanotechnology. It is the possible alternative to current CMOS technology. Reversible QCA logic is the most important issue at present time to reduce power losses. This paper presents a novel reversible logic gate called the F-Gate. It is simplest in design and a powerful technique to implement reversible logic. A systematic approach has been used to implement a novel single layer reversible Full-Adder, Full-Subtractor and a Full Adder–Subtractor using the F-Gate. The proposed Full Adder–Subtractor has achieved significant improvements in terms of overall circuit parameters among the most previously cost-efficient designs that exploit the inevitable nano-level issues to perform arithmetic computing. The proposed designs have been authenticated and simulated using QCADesigner tool ver. 2.0.3.

**Specifications Table**

**Table:** 

Subject area	*Nanoelectronics*
More specific subject area	*Nanotechnology QCA reversible logic design*
Type of data	*Table, figure*
How data was acquired	*QCADesigner software Bistable engine and Analysis process have been applied to attain the data results*
Data format	*Analyzed*
Experimental factors	*Reversible F-Gate has been proposed. It has been testified to determine various arithmetic logic circuits*
Experimental features	*Computational Simulation study has been used to determine results*
Data accessibility	*Data is available within this article*

**Value of the data**

• Gates are the basic building block to design logic in digital systems. A new reversible F-Gate has been proposed to enhance the performance of digital systems.
• Adder circuits are widely investigated since their performance can directly affect the whole digital system performance. We have proposed an optimal reversible Arithmetic circuits including Adder, Subtractor and Adder-Subtractor using the proposed F-Gate.
• The presented circuit designs and data analysis can support the researchers to reduce the circuit complexity and implement high robust Arithmetic logic designs.
• The proposed QCA reversible designs can be used to reduce hardware cost and design energy lossless arithmetic logic unit (ALU) in quantum computers.

## 1 Data

**Fig. 1:**
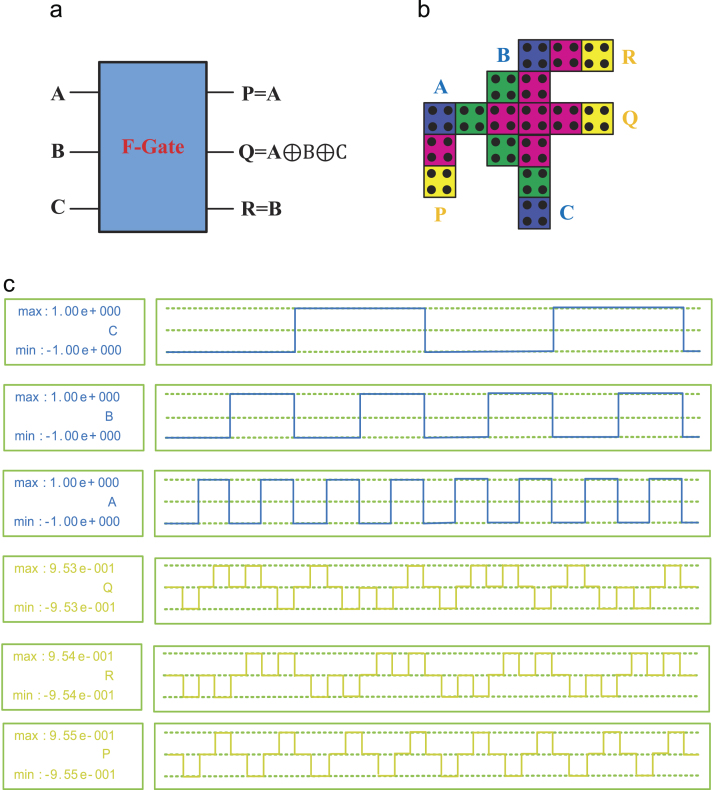
(a) Logic symbol (b) QCA Layout (c) Simulation results.

**Fig. 2:**
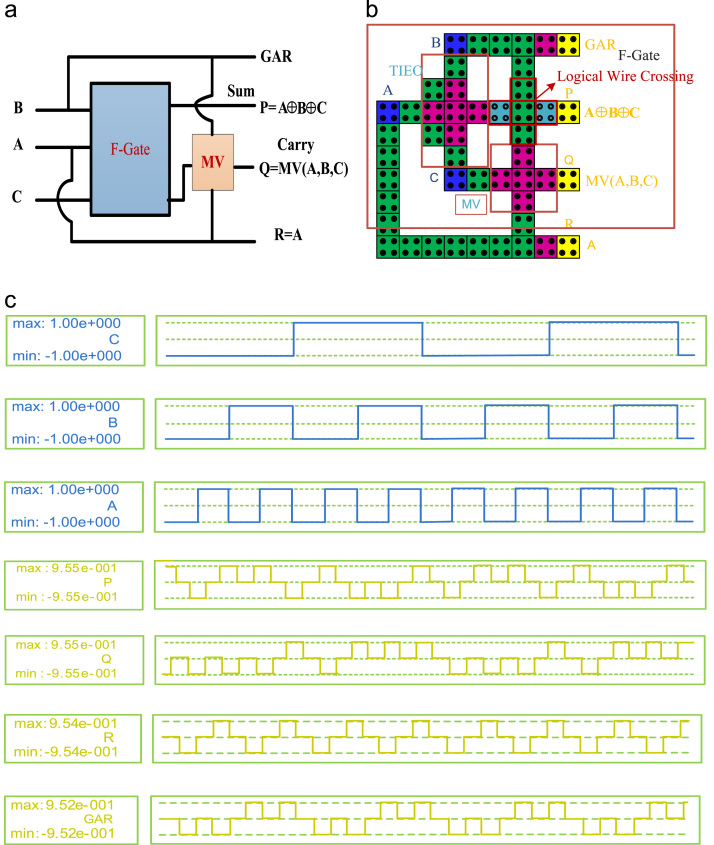
(a) Logic diagram (b) QCA Layout (c) Simulation results.

**Fig. 3:**
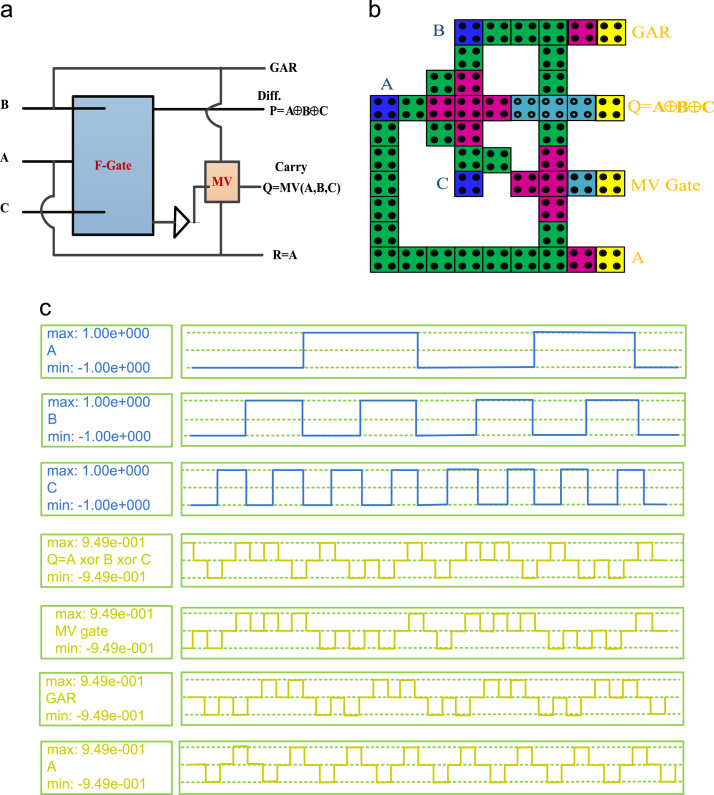
(a) Logic diagram (b) QCA Layout (c) Simulation results.

**Fig. 4:**
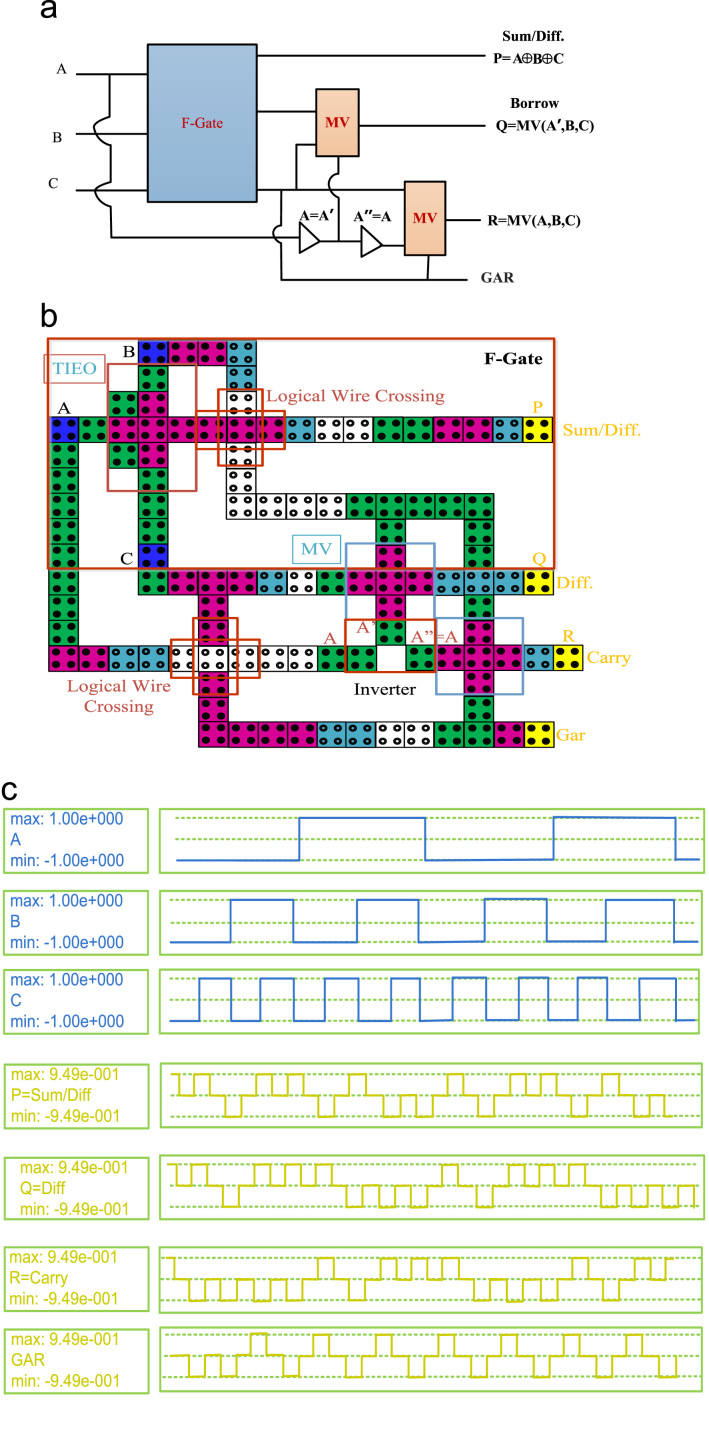
(a) Logic diagram (b) QCA Layout (c) Simulation results.

In this paper, a new high speed and a low power reversible gate called the F-Gate has been proposed. The logic symbol, QCA layout, and its simulation results are shown in Fig. 1. The proposed gate has been used in a systematic manner to implement single layer arithmetic logic functions such as reversible Full Adder (RFA), reversible Full Subtractor (RFS) and reversible Full Adder-Subtractor (RFAS). The logic symbol, QCA layout, and simulation results of the proposed Arithmetic circuits are shown in Fig. 2, Fig. 3, Fig. 4, respectively. A detailed report on the hardware costs achieved from the proposed QCA implementations in terms of area, cell counts and clock delays are provided in Table 1. However, the structural evaluation of the proposed RFAS circuit has been compared with their conventional counterparts [1], [2], [3], [4], [5]. The detailed comparison results of RFAS are shown in Table 2.

**Table 1:** QCA design characteristics.

Proposed structures	Constant-inputs	Garbage outputs	Area (μm^2^)	Circuit complexity (Cell-Counts)	Latency (Clock delays)	Cost = Area × Delay × Power
F-Gate	0	1	0.02	18	3-clock phases = (0.75)	0.27
Proposed Full Adder (RFA)	0	1	0.03	45	3-clock phases = (0.75)	1.0125
Proposed Full Subtractor (RFS)	0	1	0.04	47	3-clock phases = (0.75)	1.35

**Table 2:** Comparisons of reversible Full Adder-Subtractor circuits.

Full Adder–Subtractor designs	Constant-inputs	Garbage outputs	Area (μm^2^)	Circuit complexity (Cell-counts)	Latency (Clock delays)	Cost = Area× Delay × Power
Ref. [1]	0	3	0.46	343	6-clock phases = (1.50)	236.67
Ref. [1]	0	3	0.47	356	6-clock phases = (1.50)	250.98
Ref. [2]	1	2	0.78	517	13-clock phases = (3.25)	1310.595
Ref. [3]	3	3	0.96	612	16-clock phase = (4.0)	2350.08
Ref. [4]	0	3	0.40	351	6-clock phase = (1.50)	210.6
Ref. [5]	1	1	0.28	228	7-clock phase = (1.75)	111.72
Proposed Full Adder-Subtractor	0	1	0.11	109	7-clock phase = (1.75)	20.9825

## 2 Experimental design, materials and methods

QCADesigner tool ver. 2.0.3 [6] with default parameters have been verified the functioning of the proposed QCA-circuits. The default parameters are listed as: QCA cell size = 18 nm, diameter of quantum dots = 5 nm, number of samples = 50,000, convergence tolerance = 0.001, radius of effect = 65 nm relative permittivity = 12.9, clock low = 3.8e−23 J, clock high = 9.8e−22 J, clock amplitude factor = 2.000, layer separation = 11.5 nm and maximum iterations per sample = 100. The simulation result, shown in Figs. 1–4, validates the functionality of the proposed circuits, which has used the proposed F-Gate as its main functional block.

The construction of the F-Gate is simple in design. It consists of three inputs (*A*, *B*, & *C*) and three outputs “*P*, *Q*, & *R*”. The main processing part of the F-Gate is a three-input XOR (TIEO) [8]. The *Q* = (*A* ⊕ *B* ⊕ *C*) is carried out from the main part of the F-Gate. The logic expression of inputs & outputs are expressed as:

(1) P=A

(2) Q=(A⊕B⊕C)

(3) R=B

To testify the functionality of the F-Gate it has been used as a main component to compute Sum bits of the reversible Full-Adder (RFA), Difference of the reversible Full-Subtractor (RFS) and Sum/Difference of the reversible Full Adder-Subtractor (RFAS). Table 2 includes a comparison between our proposed Full Adder-Subtractor with their conventional counterparts. An extensive structural analysis have been developed in different aspects of Area, Circuit complexity and cost of the proposed Full Adder-Subtractor and previously published works [1], [2], [3], [4], [5]. The proposed RFAS produce one garbage outputs. However, clock zones for wire crossing signal synchronization makes the latency (number of clock cycles), a little head greater than conventional designs. But the RFAS have achieved a significant improvements in terms of Cost = Area × Delay × Power [7] than existing one. It performs both addition and subtraction operations. The main outputs of the RFAS circuit are, P = A ⊕ B ⊕ C, produces Sum/Difference. The Sum/Difference is the Sum and Difference of the three inputs (A, B, C) and Q = MV (A′, B, C) & R = MV (A, B, C) are the outputs of Borrow and Carry, respectively.
