# Supplementary material for: A novel reversible logic gate and its systematic approach to implement cost-efficient arithmetic logic circuits using QCA
Source: Data Brief. 2017 Oct 7;15:701–8. doi: 10.1016/j.dib.2017.10.011 (PMC5671476; doi:10.1016/j.dib.2017.10.011)
Supplement: Supplementary file 1 — Transparency document [file mmc1.docx]

DIBL JOURNAL

Dear Edition/ professor

Authors have declared that no competing interests exist regarding the publication of this manuscript.

Yours Sincerely.

Author Firdous Ahmad
