# Supplementary material for: A novel reversible logic gate and its systematic approach to implement cost-efficient arithmetic logic circuits using QCA
Source: Data Brief. 2017 Oct 7;15:701–8. doi: 10.1016/j.dib.2017.10.011 (PMC5671476; doi:10.1016/j.dib.2017.10.011)
Supplement: Supplementary file 2 — Supplementary material [file mmc2.docx]

DIBL JOURNAL

Dear Edition/ professor

 Fig. 1: (a) Logic symbol (b) QCA Layout (c) Simulation results

Fig. 2: (a) Logic diagram (b) QCA Layout (c) Simulation results

Fig. 3: (a) Logic diagram (b) QCA Layout (c) Simulation results

Fig. 4: (a) Logic diagram (b) QCA Layout (c) Simulation results
